# Beyond benign: ventricular ectopy as a clue to cardiac metastasis in melanoma

**DOI:** 10.1093/ehjcr/ytaf057

**Published:** 2025-02-01

**Authors:** Samah El-Mhadi, Belghait El Hajjaj, Bruno Pagis, Benjamin Safar

**Affiliations:** Cardiology Department, Intercommunal Hospital Group of Le Raincy-Montfermeil, Northeast Greater Paris 93370, France; Cardiology Department, Intercommunal Hospital Group of Le Raincy-Montfermeil, Northeast Greater Paris 93370, France; Cardiology Department, Intercommunal Hospital Group of Le Raincy-Montfermeil, Northeast Greater Paris 93370, France; Cardiology Department, Intercommunal Hospital Group of Le Raincy-Montfermeil, Northeast Greater Paris 93370, France

## Case description

Cardiac metastases often remain undetected, masked by non-specific symptoms. Malignant melanoma metastasizes to the heart in up to 64% of cases, yet cardiac involvement is diagnosed ante-mortem in less than 16%.^1^

**Figure 1:**
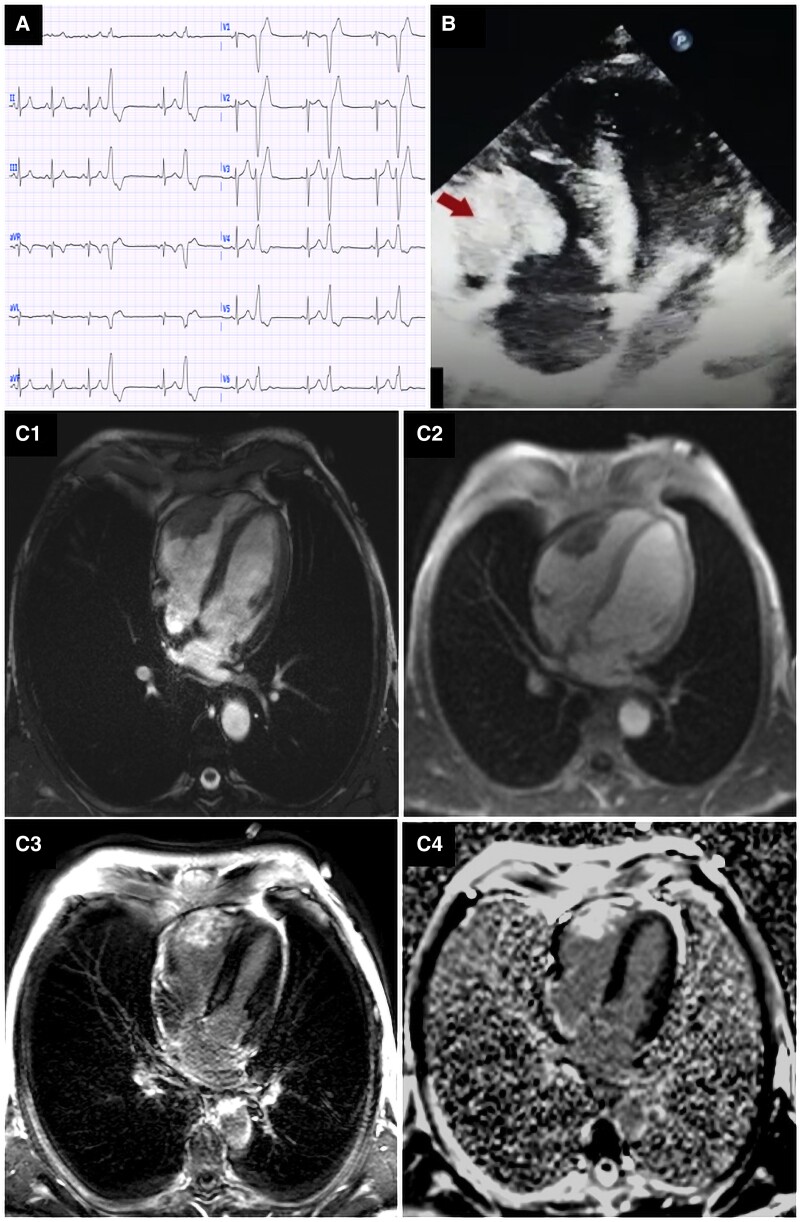
(*A*) Electrocardiogram showing premature ventricular beats with left bundle branch block morphology, organized in a bigeminy pattern. (*B*) Transthoracic echocardiography four-chamber view revealing a homogeneous and hyperechoic mass infiltrating the right ventricle’s free wall (arrow). (*C*) Cardiac magnetic resonance: cine-steady-state free precession four-chamber view showing a T2-hypointense mass (*C1*), with perfusion defects on the early perfusion sequence (*C2*), and focal late gadolinium enhancement on the 3D myocardial delayed enhancement sequence (*C3*) and phase-sensitive inversion recovery four-chamber sequence (*C4*).

We report the case of a 40-year-old male diagnosed with malignant scalp melanoma 3 years ago, who underwent surgical resection. He presented to the emergency department with resting palpitations. He was conscious and eupneic, with a blood pressure of 120/70 mmHg and a heart rate of 75 b.p.m. Cardiovascular examination was unremarkable. Resting electrocardiogram (EKG) showed sinus rhythm and premature ventricular beats (PVBs) organized in a bigeminy pattern, with left bundle branch block morphology, suggesting a right ventricular (RV) origin, most likely from the RV outflow tract (*Figure 1A*). The left ventricle was not dilated, with a preserved ejection fraction. The 24-h burden of PVB was found to be 15%. Laboratory tests showed normal D-dimer levels, haemoglobin at 13 g/dL, no electrolyte disturbances, and normal thyroid function.

A year earlier, the patient had consulted for similar symptoms and was discharged following normal biological findings and a benign interpretation of PVB on EKG.

The transthoracic echocardiography (TTE) identified moderate RV dilation (RVED area of 26 cm²) with no significant haemodynamic compromise and no regional wall motion abnormalities. A homogeneous, sessile, and hyperechoic mass was seen infiltrating the RV free wall (*Figure 1B*). Cardiac magnetic resonance confirmed a T2-hypointense mass with early perfusion defects and focal late gadolinium enhancement (*Figure 1C*) and probable insidious infiltration of the RV outflow tract. Although mapping sequences were unavailable, these findings highly suggested cardiac metastasis from melanoma.

Due to the infiltrative nature of the mass and its location, surgical resection was deemed unfeasible. Instead, a conservative approach was adopted, with clinical monitoring, regular Holter assessments for arrhythmias, and TTE to track changes in the mass.

As of 4 months post-diagnosis, the patient is clinically stable, with no significant deterioration.

Cardiac metastases are like silent predators, easily overlooked but potentially devastating. This case illustrates how subtle findings, such as ventricular ectopy initially misjudged as benign, may delay a critical diagnosis. Routine screening for cardiac involvement in high-risk melanoma patients may offer early detection, potentially improving prognosis and management.^2^ Similar cases in the literature report poor outcomes despite various interventions. Systemic therapies, such as immune checkpoint inhibitors and targeted therapies, have shown efficacy against extra cardiac metastases but limited success in controlling cardiac involvement. Symptomatic management, including antiarrhythmic drugs or implantable devices, may be necessary to address life-threatening arrhythmias. Unfortunately, survival remains poor, with most patients succumbing to widespread metastases.^3^

## Data Availability

Data sharing is not applicable as no data sets were generated or analysed for this case report.

## Notes

**Consent:** The authors confirm that written consent for submission and publication of this case report including images and associated text has been obtained from the patient in line with the Committee on Publication Ethics (COPE) guidelines.

**Funding:** None declared.
